# Effect of progesterone sequential therapy on follicle stimulating hormone (FSH), luteinizing hormone (LH), and estradiol (E2) in perimenopausal dysfunctional uterine bleeding

**DOI:** 10.5937/jomb0-53229

**Published:** 2025-06-13

**Authors:** Song Wu, Huiru Wang, Yuwei Zhao, Bo Tang, Qi Zhang, Dapeng Li

**Affiliations:** 1 Yanshan University, Post Doctoral Station of Control Science and Engineering, Qinhuangdao Hospital of Traditional Chinese Medicine, Qinhuangdao, Hebei Province, China; 2 Hebei North University, Graduate School, Zhangjiakou, Hebei Province, China; 3 The Fourth Hospital of Qinhuangdao City, Department of Internal Medicine III, Qinhuangdao, Hebei Province, China

**Keywords:** progesterone sequential therapy, perimenopausal dysfunctional uterine bleeding, clinical effect, curettage treatment, follicle-stimulating hormone, luteinizing hormone, estradiol, sekvencijalna terapija progesteronom, perimenopauzalno disfunkcionalno krvarenje iz materice, klinički efekat, tretman kiretažom, folikulostimulišući hormon, luteinizirajući hormon, estradiol

## Abstract

**Background:**

Perimenopausal dysfunctional uterine bleeding (PDUB) is a common gynaecological disease with various clinical treatment options. The objective of this work was to investigate the clinical effect of female progesterone sequential therapy (FPST) on PDUB and follicle-stimulating hormone (FSH), luteinizing hormone (LH), and estradiol (E2).

**Methods:**

140 cases of PDUB patients were enrolled and randomly rolled into an observation (Obs) group and a control (Ctrl) group, with 70 cases in each. The patients in the Ctrl group were given pure curettage treatment, and those in the Obs group were supplemented with FPST based on the intervention in the Ctrl group.

## Abstract (sr)

**Uvod:**

Perimenopauzalno disfunkcionalno krvarenje iz materice (PDUB) je česta ginekološka bolest sa raznovrsnim kliničkim mogućnostima lečenja. Cilj ovog rada bio je da se istraži klinički efekat sekvencijalne terapije ženskog progesterona (FPST) na PDUB, kao i na folikulostimulišući hormon (FSH), luteinizirajući hormon (LH) i estradiol (E2).

**Metode:**

U studiju je uključeno 140 pacijentkinja sa PDUB-om, koje su nasumično podeljene u posmatranu (Obs) grupu i kontrolnu (Ctrl) grupu, sa po 70 pacijentkinja u svakoj grupi. Pacijentkinje u Ctrl grupi su podvrgnute isključivo kiretaži, dok su pacijentkinje u Obs grupi, na bazi intervencije iz Ctrl grupe, dobile i sekvencijalnu terapiju progesteronom (FPST).

**Rezultati:**

Upoređeni su terapeutski efekti kod pacijentkinja u različitim grupama. Stopa izlečenja (CR) i ukupna stopa efikasnosti (TER) u Obs grupi su iznosile 84,26% i 97,14%, što je znatno više nego u Ctrl grupi (44,29% i 74,29%), što ukazuje na značajne razlike (P<0,05). Incidenca neželjenih reakcija u Obs grupi i Ctrl grupi iznosila je 4,29% (3/70) i 2,86% (2/70), respektivno, ne pokazujući značajnu razliku (P>0,05). Pored toga, nakon tromesečnog praćenja, stopa recidiva u Obs grupi je iznosila 1,43% (1/70), što je bilo znatno niže u poređenju sa 44,29% (31/70) u Ctrl grupi, što je predstavljalo značajnu razliku sa P<0,05.

**Zaključak:**

Primena sekvencijalne terapije progesteronom (FPST) uz kiretažu kod PDUB-a može efikasno da poboljša klinički efekat lečenja i smanji stopu recidiva.

## Introduction

Perimenopause is the female reproductive system gradually from the fertile period to the menopause transition stage, which is an important physiological transition period in female life [1]. Among them, dysfunctional uterine bleeding is one of the common symptoms of perimenopause, mainly manifested as irregular menstrual volume, irregular cycle, prolonged menstruation, and other symptoms [2]. Perimenopausal women’s abnormal uterine bleeding affects quality of life; hysteroscopic treatments offer alternatives to hysterectomy with varied outcomes [3] [4]. Perimenopausal women with abnormal uterine bleeding require treatment evaluation, with urgency varying from anaemia to potential cancer diagnosis consideration. Treatment methods include estrogen replacement therapy, oral contraceptives, intrauterine devices, etc., but there are certain side effects and limitations [3] [4]. Estrogen-progesterone, as a commonly used hormone replacement therapy, has been widely used in perimenopausal women. By supplementing estrogen and progesterone, estrogen and progesterone can adjust endocrine levels, thus alleviating perimenopausal symptoms and improving quality of life [5].

Moreover, it has been applied to a certain extent in clinical practice as a comprehensive treatment of different types of hormones. However, its curative effect in PDUB is not yet clear [6]. Ismet Inan et al. found tibolone and estrogen-progestogen therapy equally effective in alleviating perimenopausal psychological symptoms, with added lipid benefits [7]. Jahedbozorgan & Hasanzadeh found that continuous hormone therapy led to amenorrhea in postmeno pausal women, whereas sequential therapy resulted in varied bleeding [8]. Armeni et al. [9] recommend se quential hormone therapy for managing meno pause, with individualization key to minimizing risks and maximizing efficacy benefits [10]. Ruan & Mueck reviewed and recommended sequential-combined estrogen/progestogen regimens for optimizing menstrual regulation and minimizing risks, with transdermal estradiol and progesterone or dydrogesterone as the »golden standard« for reducing venous thromboembolism and stroke risks in menopausal hormone therapy.

Despite the existing literature on the use of hormone replacement therapy (HRT) in perimenopausal women, there are several deficiencies in the current understanding of its effectiveness in managing PDUB. Firstly, most studies have focused on the use of estrogen-progesterone therapy in postmenopausal women, with limited research on its application in perimenopausal women with PDUB [8] [9]. Secondly, the existing studies have primarily evaluated the effects of HRT on menopausal symptoms, such as hot flashes and night sweats, with few studies examining its impact on PDUB specifically. Furthermore, the optimal regimen and dosage of HRT for managing PDUB remain unclear [8] [9] [10]. Therefore, this study aimed to investigate the clinical effect of female progesterone sequential therapy (FPST) on PDUB, focusing on its impact on FSH, LH, and estradiol levels. By comparing the therapeutic effects of FPST with those of pure curettage treatment, this study aimed to provide new insights into managing PDUB and contribute to developing more effective treatment strategies for perimenopausal women. Ultimately, this study aims to inform the development of more effective and personalized treatment strategies for perimenopausal women with PDUB, improving their quality of life and reducing the risk of complications associated with this condition.

## Materials and methods

PDUB patients admitted to Qinhuangdao Hospital of Traditional Chinese Medicine from February 2022 to December 2023 were enrolled and randomly rolled into an observation (Obs) group and a control (Ctrl) group, with 70 cases each. This work has been approved by the Medical Ethics Committee of Qinhuangdao Hospital of Traditional Chinese Medicine (with code of 2014379), and all the patients’ families participating in the study have signed consent forms.

The patients enrolled here had to satisfy all the following items: ① perimenopausal women, aged 45–55 years; ② there were clinical symptoms of dysfunctional uterine bleeding including menorrhagia, irregular menstruation, and prolonged menstruation; and ③ no obvious organic diseases such as uterine fibroids and endometriosis were found through clinical examination and B-ultrasonography. The patients had to be excluded if they had any of the below conditions: ① patients with liver, kidney, and other organ diseases and those who had taken hormone drugs in the past 3 months; ② patients with a history of drug allergy related to this study; and ③ blood system diseases, such as coagulation dysfunction, were detected.

### Treatment methods

Pure curettage treatment was given to patients in the Ctrl group. FPST treatment, including sequential use of estrogen and progesterone, was given to patients in the Obs group. That is, synthetic estrogens, such as estradiol, taken orally at 1 mg daily during the first 14 days of the menstrual cycle; using a synthetic progesterone, such as medroxyprogesterone acetate, orally at 10 mg daily on days 15–28 of the menstrual cycle.

### Evaluation criteria and observation indexes of efficacy

The treatment efficacy was evaluated as four degrees. Cured: the symptoms of irregular vaginal bleeding disappeared completely, and there was no recurrence in 6 months. Obviously effective: the menstrual cycle and blood volume returned to normal or amenorrhea, and there was no recurrence at 6 months. Effective: the menstrual cycle and blood volume returned to normal. Ineffective: the menstrual cycle did not change, and the symptoms of vaginal bleeding did not improve or worsen compared with before treatment. After treatment, 5 mL of fasting venous blood was collected, centrifuged at 1,500 rpm for 10 min, and serum was collected to measure the follicle-stimulating hormone (FSH), luteinizing hormone (LH), and estradiol (E2) by radioimmunoassay. All patients were followed up for 3 months to count the recurrence and adverse reactions for comparison.

### Statistical method

SPSS18.0 was utilized for statistical analysis of the data. The count and measurement data were expressed by frequency and mean ± standard deviation, respectively, subjecting to the 2 and independent sample t-tests. *P*<0.05 meant a statistically significant difference.

## Results

**Figure 1:**
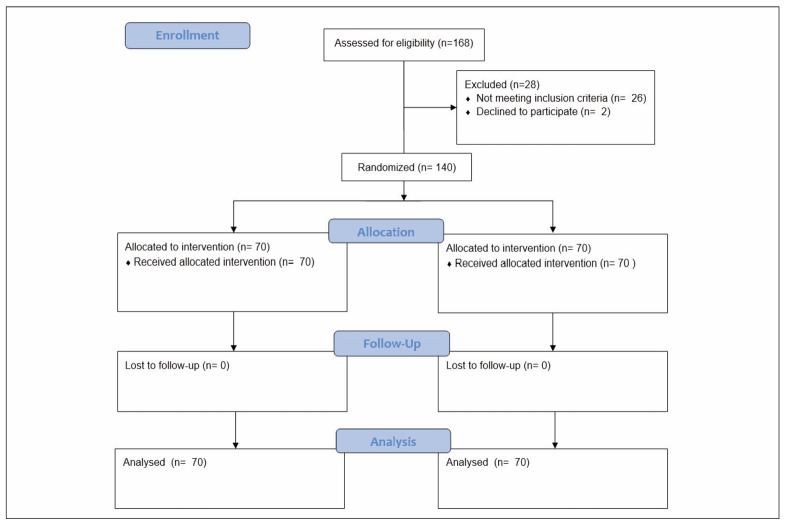
CONSORT flow diagram of the study.

The final number of 140 patients was included, as illustrated in Figure 1.

In the Obs group, the patients were 45~50 years old and (47.5±2.8) years old. The course of disease (COD) ranged from 4 to 13 months ((6.7±1.2) months on average), the heart rate (HR) was (76.5±5.6) beats/min, the systolic blood pressure (SBP) was (115.3±9.5) mmHg, and the diastolic blood pressure (DBP) was (76.1±6.7) mmHg. The 70 patients in the Ctrl group ranged in age from 44 to 50 years ((47.1±3.2) years on average). The COD was 4∼12 months (averaged as (6.6±1.3) months). Meanwhile, the HR, SBP, and DBP were (77.1±6.0) beats /min, (116.0±8.8) mmHg, and (77.3±7.0) mmHg, respectively. A comparison of the above data revealed that the difference was insignificant (*P*>0.05).

Table 1 illustrates the clinical efficacies of patients in different groups. In the Obs group, 59 patients were cured, 5 were obviously effectively treated, 4 were effectively treated, and 2 were ineffectively treated. In the Ctrl group, the numbers of patients with cured, obvious effective, effective, and ineffective efficacy were 31, 10, 11, and 18, respectively. The above data suggested that the CR and TER in the Obs group (84.26% and 97.14%) were greatly higher than those in the Ctrl group (44.29% and 74.29%), exhibiting obvious differences (*P*<0.05). The specific data of CR and TER are displayed in Table 1.

**Table 1:** Comparison of Clinical Data and Outcomes between Obs and Ctrl Groups.

Variable	Obs Group (n=70)	Ctrl Group (n=70)	P-value
Age (years)	47.5±2.8 (45–50)	47.1 ± 3.2 (44–50)	0.42
The course of disease (months)	6.7±1.2 (4–13)	6.6 ± 1.3 (4–12)	0.63
Heart Rate (beats/min)	76.5±5.6	77.1±6.0	0.51
Systolic Blood Pressure (mmHg)	115.3±9.5	116.0 ±8.8	0.71
Diastolic Blood Pressure (mmHg)	76.1±6.7	77.3±7.0	0.55
Clinical Efficacy			
‣ Cured	59	31	<0.001
‣ Obviously Effective	5	10	0.03
‣ Effective	4	11	0.24
‣ Ineffective	2	18	<0.001
Cure Rate (%)	84.26	44.29	<0.001
Total Effective Rate (%)	97.14	74.29	0.02
FSH (U/L)	22.8±3.6	35.1±4.2	<0.001
LH (U/L)	21.1±2.8	29.6±3.7	<0.001
E2 (pmol/L)	120.4±5.9	185.5±6.7	<0.001
Adverse Reactions (%)	4.29 (3/70)	2.86 (2/70)	0.68
Recurrence Rate (%)	1.43 (1/70)	44.29 (31/70)	<0.001

The FSH, LH, and E2 levels of patients after they were treated differently were compared in Table 1. The above three indicators in the Obs group were (22.8±3.6) U/L, (21.1±2.8) U/L, and (120.4±5.9) pmol/L, respectively; while those in the Ctrl group were (35.1±4.2) U/L, (29.6±3.7) U/L, and (185.5±6.7) pmol/L, respectively. These findings suggested that the FSH, LH, and E2 levels of patients treated by FPST with curettage treatment were greatly lower than those treated by pure curettage treatment, showing obvious differences with *P*<0.05.

Among the 70 patients in the Obs group, 3 cases had mild adverse reactions such as nausea and vomiting after taking the drug, which disappeared without clinical treatment, and the incidence of adverse reactions was 4.29% (3/70). In the Ctrl group, 2 patients had mild adverse reactions such as nausea and vomiting after taking the drug, which disappeared without clinical treatment, and the incidence of adverse reactions was 2.86% (2/70). The comparison in incidences of adverse reactions of patients receiving different treatments exhibited no remarkable difference (*P*>0.05), as illustrated in Table 1.

After 3 months of follow-up, of the 70 patients in the Obs group, only 1 patient had recurrence, with a recurrence rate of 1.43% (1/70), while 31 patients had recurrence, with a recurrence rate of 44.29% (31/70) in the Ctrl group. As demonstrated in Table 1, the patients in the Obs group after the FPST with curettage treatment presented a lower recurrence rate than those treated by pure curettage treatment (*P*<0.05).

## Discussion

PDUB is a common gynaecological disease, and there are many clinical treatment options, among which FPST has been widely undertaken as a conservative treatment [11] [12] [13]. The results of this work suggested that FPST has an obvious curative effect in treating PDUB. First, patients in the Obs group showed great improvement in menstrual cycle and menstrual volume. This work revealed that the TER and CR of patients after the FPST with curettage treatment were 97.14% and 84.26%, while those for patients treated with pure curettage were 74.29% and 44.29%, respectively. This suggests that FPST can regulate the menstrual cycle and reduce menstrual volume by adjusting estrogen levels, thus alleviating the symptoms of PDUB. This is consistent with the findings of many previous studies, which verified the effectiveness of FPST in treating PDUB [14] [15]. All patients had no serious adverse reactions, and patients with mild adverse reactions were slightly more in the Obs group, but *P*>0.05. The incidence of adverse reactions in patients after the FPST with curettage treatment was 4.29% (3/70), and that was 2.86% (2/70) for patients who were treated with pure curettage treatment, showing no great difference (*P*>0.05). This indicates that FPST is relatively safe in clinical application, and adverse reactions are mild and tolerable [16]. However, it should be noted that the sample size enrolled herein was small, and the treatment duration was short. Further, large-scale and long-term follow-up studies are needed to fully evaluate its long-term safety [17].

As a conservative therapy for treating PDUB, FPST has significant clinical effects [18]. By adjusting estrogen levels, the menstrual cycle and menstrual volume can be improved, thus relieving symptoms of PDUB and improving the QOL of patients [19] [20]. Secreted by the pituitary gland, FSH can promote the increase of endogenous LH and E2 levels, thus leading to the imbalance of estrogen and progesterone levels. It was indicated that the FSH, LH, and E2 levels greatly decreased after different interventions, and the degree of decrease was more obvious after FPST treatment. These results indicate that FPST can regulate hormone imbalance in PDUB patients. In addition, FPST showed good safety and tolerance in this study. Compared with traditional hormone replacement therapy, FPST has the advantage that it can better simulate the natural menstrual cycle and avoid the discomfort and side effects that may be caused by continuous hormone replacement [21] [22].

Additionally, FPST uses the alternate application of estrogen and progesterone, avoiding hormone dependence and decreased tolerance that can result from the long-term use of a single hormone. This gives an ideal choice for clinical treatment. However, this work was subject to several limitations [23]. In clinical practice, FPST has shown good efficacy and safety in treating PDUB [24] [25]. However, more large-scale and long-term follow-up clinical studies are still needed to confirm its efficacy and safety further and compare it with other treatment methods that can better guide clinical practice [26] [27] [28]. Further research and discussion are needed on the individualized treatment plan for different patients, including the type, dosage, and course of estrogen-progesterone.

To discuss the physiological aspects of the effects seen by this treatment, the menstrual cycle is regulated by the hormones estrogen (estradiol) and progesterone (medroxyprogesterone acetate). During days 1–14, estradiol stimulates the growth and thickening of the uterine endometrium by binding to estrogen receptors, leading to increased cell proliferation, angiogenesis, and glycogen and lipid synthesis. From days 15–28, progesterone promotes differentiation and preparation of the endometrium for implantation by binding to progesterone receptors, leading to changes in gene expression, decidualization, and the production of prostaglandins. If pregnancy does not occur, the withdrawal of progesterone on days 29-30 leads to the shedding of the endometrium, resulting in menstruation. This complex interplay between estrogen and progesterone regulates various biological processes, including endometrial growth, angiogenesis, immune modulation, and withdrawal bleeding, and is essential for a healthy menstrual cycle [29].

## Conclusion

In summary, supplementing with FPST in the process of implementing curettage treatment of PDUB can effectively improve clinical effects and reduce the recurrence rate, which is worthy of in-depth clinical research and promotion. Due to the small sample size and research limitations, further large samples were still needed to verify and improve this conclusion. Future studies should be conducted to explore the therapeutic mechanism of this therapy and apply it to clinical practice to assess its safety and efficacy more fully.

## Dodatak

### Funding

The research is supported by Wenyang stickers on spleen and kidney yang deficiency chronic bronchitis action mechanism research project number: 2014379, Patent Number: ZL 2015 1 0250209.8.

### Conflict of interest statement

All the authors declare that they have no conflict of interest in this work.
